# Extracellular Vesicles in Comorbidities Associated with Ischaemic Heart Disease: Focus on Sex, an Overlooked Factor

**DOI:** 10.3390/jcm10020327

**Published:** 2021-01-17

**Authors:** Claudia Penna, Saveria Femminò, Giuseppe Alloatti, Maria F. Brizzi, Tommaso Angelone, Pasquale Pagliaro

**Affiliations:** 1 Department of Clinical and Biological Sciences, University of Turin, Regione Gonzole 10, 10043 Orbassano (TO), Italy; pasquale.pagliaro@unito.it; 2 Department of Medical Sciences, University of Turin, Corso Dogliotti 14, 10126 Turin, Italy; mariafelice.brizzi@unito.it; 3 Uni-Astiss, Polo Universitario Rita Levi Montalcini, 14100 Asti, Italy; giuseppe.alloatti@unito.it; 4 Laboratory of Cellular and Molecular Cardiovascular Pathophysiology, Department of Biology, E. and E.S. (Di.B.E.S.T.), University of Calabria, 87036 Rende (CS), Italy; tommaso.angelone@unical.it

**Keywords:** extracellular vesicles, metabolic syndrome, diabetes, hypertension

## Abstract

Extracellular vesicles (EV) are emerging early markers of myocardial damage and key mediators of cardioprotection. Therefore, EV are becoming fascinating tools to prevent cardiovascular disease and feasible weapons to limit ischaemia/reperfusion injury. It is well known that metabolic syndrome negatively affects vascular and endothelial function, thus creating predisposition to ischemic diseases. Additionally, sex is known to significantly impact myocardial injury and cardioprotection. Therefore, actions able to reduce risk factors related to comorbidities in ischaemic diseases are required to prevent maladaptive ventricular remodelling, preserve cardiac function, and prevent the onset of heart failure. This implies that early diagnosis and personalised medicine, also related to sex differences, are mandatory for primary or secondary prevention. Here, we report the contribution of EV as biomarkers and/or therapeutic tools in comorbidities predisposing to cardiac ischaemic disease. Whenever possible, attention is dedicated to data linking EV to sex differences.

## 1. Introduction

Ischaemic diseases include pathological conditions connoted by vascular disease and reduced tissue blood supply. In particular, myocardial ischaemia occurs when a partial or complete coronary artery obstruction occurs and coronary blood flow is reduced and/or not adequate for the myocardial needs.

Peripheral and coronary artery diseases are usually associated with chronic comorbidities, such as metabolic syndrome, including hypertension, obesity, and diabetes, which affect the endothelial functions of both the macro and microvasculature [1,2,3,4].

Of course, ischaemia, if untreated, can lead to tissue death [5]. Actually, starting from the 1970s, experimental studies of reperfusion/revascularization in animals and thrombolysis and percutaneous coronary intervention (PCI) in humans have shown that revascularization/reperfusion not only reduces the infarct size but also allows a better recovery from the myocardial contractile dysfunction [6,7]. Furthermore, coronary artery bypass surgery and subsequently elective PCI in patients with chronic stable angina became feasible [8]. Therefore, the benefits of reperfusion/revascularization have become evident. However, over the years, we have realised that reperfusion is not entirely effective, but alone, it led to pathophysiological phenomena. Indeed, revascularization and reperfusion may add damage to a tissue already jeopardised by the ischaemia itself. At the same time, we became aware that myocardial ischaemia cannot simply represent a negative process since it can enable the adaptation mechanisms of conditioning [9,10]. Recent studies have questioned the superiority of revascularization/reperfusion over medical therapy in improving symptoms and prognosis of patients with chronic coronary syndromes [11,12]. Therefore, nowadays, the most effective “treatment” for all vascular ischaemic diseases is to reduce and control the risk factors related to these conditions, such as smoking, high blood pressure, and high cholesterol. Ideally, primary and secondary prevention are necessary to reduce the burden of cardiovascular- and ischaemic-related diseases [13,14]. Hopefully, EV may be future early biomarkers and therapeutic tools to limit vascular inflammation that plays a pivotal role in initiating and maintaining atherosclerotic and ischaemic diseases, both in men and women. Indeed, the regulation of inflammation may ameliorate both morbidity and mortality in both sexes [14].

**Figure 1:**
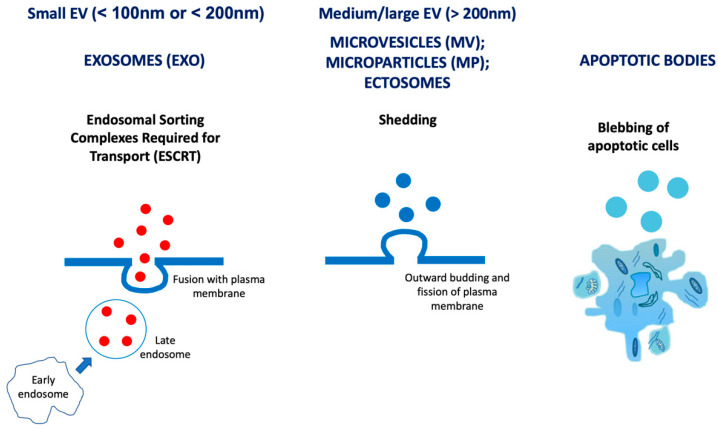
Classification of extracellular vesicles (EV) into “small” and “medium/large”. Small EV (sEV; <100 nm or <200 nm) originate by a mechanism of endosomal sorting complexes required for transport (ESCRT) machinery; medium/large EV (m/l EV; >200 nm) originate from the shedding after membrane budding or by blebbing of apoptotic cells [16].

Recently, circulating extracellular vesicles (EV) have been recognised as prognostic and therapeutic tools for several pathological conditions, including cardiovascular diseases (CVDs) [15]. EV are a heterogeneous population of cell-derived membranous structures. According to according to the guidelines of the International Society for Extracellular Vesicles (ISEV), on Minimal Information for Studies of EV (MISEV), EV can be classified based on their physical characteristics, such as size, in “small EV” (sEV; <100 nm or <200 nm) and “medium/large EV” (m/l EV; >200 nm) or density (low, middle, high, with each range defined) [16]. Small EV originate by a mechanism of endosomal sorting complexes required for transport (ESCRT) machinery; medium/large EV originate from the shedding after membrane budding or by blebbing of apoptotic cells (Figure 1) [17].

As reported in [15,18], the generic term “extracellular vesicle” refers to all particles released from cells and delimited by a lipid bilayer, which have priority over the term before others, such as exosomes, which represent only EV generated by the endosomal system [18]. Several subtypes are defined based on their biochemical and physical characteristics and in particular their cell origin, molecular markers, size, density, and function. Absolute EV isolation and purification or complete isolation is a complex goal, but several strategies or combinations of strategies have been or are currently being developed [15,18]. Of note, small EV/exosomes are completely different from microparticles (MV)/monocytes (MP)/ectosomes, and often have different functions. Moreover, MV, especially platelet-derived MV, are often pro-thrombotic. Therefore, whenever possible, we distinguish between small EV/exosomes and larger EV/MV or MP.

All EV are released from all cell types and are detectable in many body fluids. Although for a long time EV were considered only as double-layer phospholipid membrane vesicles and not biologically significant particles, they have emerged as biological entities able to influence the behaviour of target cells [19]. The effect of EV depends not only on their origin but also on the microenvironment containing the donor cells [20,21,22]. Due to their ability to carry their cargo, consisting of proteins, lipids, aminoacids, mRNAs, and miRNAs, circulating EV can be exploited as promising biomarkers for comorbidities of ischaemic conditions [23,24]. In addition to affecting the susceptibility of the myocardium to the ischaemia/reperfusion insult and the efficacy of classical cardioprotective interventions such as ischaemic conditioning [10,25], comorbidities may impact EV characteristics, modulating their composition, abundance, and function.

Recently, a number of reviews analysed the role of EV (Exo, MV, and MP) in the cardiovascular system, as biomarkers, cardioprotective, or deleterious agents [15,26,27,28]. However, very few studies and reviews considered the sex differences in terms of EV in ischaemic diseases and their correlated comorbidities. It has been established that the responses to pharmacological agents as well as to the application of cardioprotective manoeuvres are influenced by sex and comorbidities [29,30,31]. Therefore, it is important to consider the possibility of personalised medicine that will take into account sex differences, which can also be related to specific comorbidities. Here, we aim to analyse the abundance, composition, and protective efficacy of EV in the presence of comorbidities (such as metabolic syndrome, obesity, diabetes, or hypertension) and sex. This is of particular relevance since these conditions may act as potential confounders if EV were used as both biomarkers and cardioprotective tools.

## 2. Sex-Specific Differences of EV in Healthy Humans

Before considering the role of comorbidities, we briefly review studies that have considered sex-specific differences of EV in healthy humans. Studies have shown that circulating EV of different origins are more abundant in healthy women than in healthy men of the same age [32]. A correlation has been proposed between hormonal influence and the abundance of circulating EV in women. In particular, during the luteal phase of the menstrual cycle, a significant increase in circulating MP has been detected, when compared to men [33]. However, Bammert et al. [34] noticed that in middle-aged, healthy men and women, no differences in the levels of circulating MP can be detected. By contrast, they found sex-related differences in miRNA EV content. Notably, miR-125a expression was lower and miR-34a higher in the EV of men than in women. These results suggest that the number and composition (e.g., miRNA expression) of EV are sex-dependent in healthy humans.

During pregnancy, a hormonal storm occurs and EV may be strongly influenced. Indeed, EV in pregnancy are gaining significant attention in the literature (for review see [35]). However, we are not aware of studies that have analysed the role of pregnancy-related hormones, such as oxytocin and relaxin, on EV functioning. Actually, oxytocin and relaxin are sex-related hormones with cardiovascular effects. The former is the major breastfeeding hormone and displays cardioprotective properties through the activation of pro-survival pathways [36]. The latter is a pregnancy-related hormone which exerts multiple beneficial effects, including limitation of arrhythmias and inflammation and reversal of fibrosis after myocardial infarction [37]. Nevertheless, hearts of pregnant rats are more prone to ischemia/reperfusion damage compared to non-pregnant animals [37], a finding in line with clinical data [38]. Yet, EV derived from amniotic fluid display cardioprotective properties [39]. These scant and apparently discordant results suggest that further studies are necessary on the role of hormones in EV physiology.

To date, no human or animal studies have compared the cardioprotective effectiveness of EV from men/males or from women/females in clinical or experimental settings. Indeed, EV can exert effects on the heart through multiple pathways [15,28]; however, to our knowledge, no studies have evaluated whether their targets depend on sex difference.

All in all, sex differences in the numbers of EV produced, type of EV produced (or cargo) are starting to emerge. However, data lack completely regarding the possible differences in effectiveness of EV derived from males or females.

## 3. EV as Biomarkers of Ischaemic Disease-Associated Comorbidities

Comorbidities associated with ischaemic diseases, which include hypertension, dyslipidaemia, obesity, hypertension, and glucose intolerance, are linked and mutually dependent [30,31,32,33,34,35,36,37,38,39,40]. It has been suggested that metabolic disorders favour change in EV release in terms of abundance, cell origin, and composition, thus representing a valuable tool of disease prediction [41]. Differences in adipose distribution and steroid hormones between women and men translate into a different metabolic risk. Indeed, the typical female adipose distribution is associated with a lower cardiometabolic risk [30,42]. Therefore, sex-related hormonal differences might modify EV release and behaviour due to a differential cargo composition also in pathological conditions. Yet, we are of the opinion that it is recommendable to consider the role of EV in studying the role of miRNAs, as EV contain hundreds of miRNAs. For instance, inhibition of miRNA-34a (an EV cargo), which is implicated in cardiac myocardial damage, significantly attenuates cardiomyopathy-induced morphological variations in female mice compared to male mice [43]. Nevertheless, this study did not consider the role of EV. Of note, other conditions may also affect EV characteristics and function. For instance, off-pump coronary artery bypass (OPCAB) surgery displays a significant effect on the procoagulant activity of MP as well as on tissue factor and protein Z system [44].

**Figure 2:**
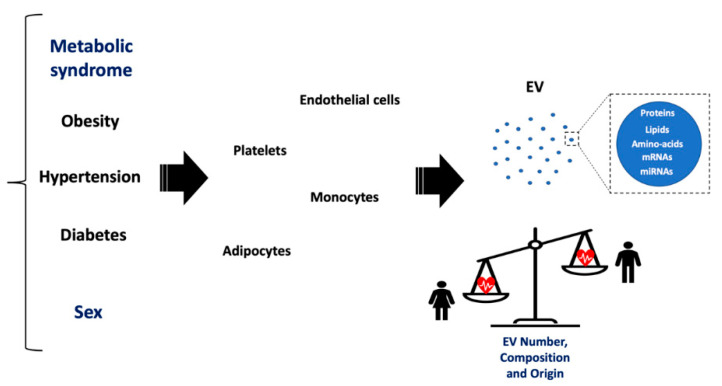
EV involvement in metabolic syndrome and sex. In comorbidities predisposing to cardiac ischaemic disease, such as metabolic syndrome (obesity, diabetes, hypertension), EV are mainly released by platelets, endothelial cells, monocytes, and adipocytes. Based on sex differences, the EV number, composition and origin could be influenced by these pathological conditions. The balance points towards women because female sex has fewer cardiovascular risk factors and different EV number and composition. Moreover, EV of different origins are more abundant in healthy women than in healthy men of the same age [42].

Since EV may be considered novel biomarkers of diabetes and other CVDs [20,45,46], discrete sex-dependent EV cargo differences, due to hormonal modulation, should be considered as they may help to explain the different risks of developing ischaemic conditions in men and women. In this regard, EV are certainly promising biomarkers of comorbidities for cardiac ischaemic conditions [23,24]; however, little attention has been paid to sex differences (Table 1). Thereby, the behaviour of EV in the different ischaemic disease comorbidities will be discussed in detail and sex differences discussed whenever possible (Figure 2).

## 4. EV and Metabolic Syndrome

Metabolic syndrome is a complex condition characterised by some clinical parameters, including raised systemic blood pressure, low high-density lipid-cholesterol, increased triglyceride levels, increased waist circumference, diabetes, and hyperglycaemia [10,47,48]. Moreover, it has been reported that the onset of atherosclerosis is more pronounced in females with metabolic syndrome. High-density lipoprotein cholesterol showed the strongest impact on intima-media thickness in men, whereas blood glucose ranked first in women [49].

In this context, sex may represent a confounder that must be kept in mind to elucidate disease-specific pathways in metabolic diseases. In this regard, Kobayashi et al. [50] demonstrated that circulating EV number is closely associated with sex and some metabolic parameters, such as triglycerides, glucose, or total cholesterol levels. They also reported that an increased EV number can be detected in men with elevated triglyceride levels, compared to both healthy men without risk factors and to women with metabolic risk [50]. A different study revealed that, in obese mice and humans, elevated levels of circulating adipocyte-derived EV are common. Additionally, reduced levels of circulating EV were found after a reduced-calorie procedure in obese subjects [51]. An increased level of circulating endothelial- and platelet-derived MP was found in obese women compared with thin women of similar age [52,53]. A raised level of serum endothelial-derived MP was detected in women with high body mass index and polycystic ovary syndrome, a condition associated with metabolic syndrome [54].

Increased circulating EV levels, especially MP, were also found in metabolic disorders, such as obesity, diabetes mellitus, and hypertension, both in animal models and in humans [55,56]. Although these conditions represent a cluster of interconnected risk factors, we attempt to separately consider how such conditions drive EV features. Although very few studies provided sex disaggregated data (Table 1), whenever possible, we underlined those studies which considered this important factor.

### 4.1. EV and Obesity

To study the involvement of EV in obesity, attention has been focused on the levels, the origin, and the action of EV (for extensive reviews, see [56,57,58,59,60]). Here and in Table 1, we summarize the main findings.

It appears that the number of circulating EV is dynamic and related to changes in body weight. Higher levels (about 10-fold) of plasma EV have been reported in overweight patients showing excessive body mass index (BMI), increased waist circumference, and augmented fat tissue mass, when compared with healthy subjects with normal weight [50,61]. The idea that EV can be also involved in obesity-associated comorbidities is supported by homeostatic model assessment for insulin resistance and by the positive correlation between MP number and the augmented fasting insulin level [62].

By using specific markers to investigate the origin and content of EV in obese patients [53,62] or in Exo derived from adipocyte primary cell culture [63], it has been suggested that increased EV levels in obesity are related to the metabolic state of adipocytes, platelets, leukocytes, hepatocytes, and endothelial cells. Indeed, lower plasma levels of endothelial-, platelet-, and leukocyte-derived MP have been detected in obese patients subjected to sleeve gastrectomy [62] or after three months of diet and exercise [59,64]. Exercise is an interesting way to increase circulating EV with protective effects against myocardial ischemia/reperfusion damage and, in the future, it may serve as a therapy against myocardial damage [65]. Moreover, a caloric restriction was effective in reducing the level of adipocyte-derived EV in obese patients [51]. Similar results were obtained in animal models of obesity, showing increased circulating level of EV derived from tissues involved in metabolic syndrome and obesity, such as the endothelium, the adipose tissue, and the immune cells [51,59].

Obesity has also been shown to alter the mRNA, miRNA, proteins, and adipokines cargo in Exo derived from adipose tissue [63,66]. In addition, MV isolated from plasma of obese patients and metabolic syndrome have been recently identified as specific carriers of Macrophage Migration Inhibitory Factor (MIF). MIF actions mainly rely on ERK activation, whose pathway has been involved in the development of insulin-resistance associated with diabetes and obesity. This suggests that the MIF-pathways should be reconsidered with regard to EV-associated proteins [61].

In view of the central role of adipose tissue as a regulator of systemic energy homeostasis, particular attention has been devoted to adipose tissue-derived EV. Exo derived from obese subject-derived adipose tissues displayed an altered expression of key proteins involved in signalling pathways related to inflammation, such as Wntβ-catenin and Transforming growth factor beta (TGF-β). Intriguingly, these adipocyte-derived Exo can influence gene expression in the A549 cell line (epithelial cells derived from human Caucasian lung carcinoma) [63]. Moreover, impaired insulin signalling has been observed in the hepatocyte cell line HepG2 after incubation with EV released from overweight or obese subject-derived adipose tissue [67]. These findings have been confirmed in animal models. Injection of adipose tissue-derived small EV (Exo-like) from obese mice induced a pro-inflammatory state in lean mice, enhancing Tumour Necrosis Factor α (TNFα) and Interleukin-6 (IL-6) circulating levels, glucose intolerance, and insulin resistance [68]. In addition, EV derived from obese patients or obese animal-derived adipose tissue induced the activation of peripheral blood mononuclear cells towards macrophages with a pro-inflammatory phenotype (M1 macrophages) [67,68]. The generation of an M1 phenotype resulting from the lipid increase in adipocytes may result in a higher M1 macrophage infiltration and translates into insulin resistance in lean mice [68].

Insulin resistance associated with obesity may also depend on the activation of peripheral mononuclear cells by adipose tissue-derived small EV (Exo-like) [68]. Indeed, in vitro experiments showed that treatment of adipocytes with a conditioned medium of macrophages activated by adipose tissue-derived EV from obese patients impairs insulin signalling [67]. These small EV derived by adipose tissue of high-fat diet mice induced inflammation and insulin resistance in lean animals [68]. In addition, the level of EV derived from subcutaneous adipose tissue resulted inversely correlated with waist circumference and the presence of metabolic syndrome complications. Moreover, EV released from the omental adipose tissue were positively correlated with plasma liver enzymes [67,69]. Of note, EV derived from adipose-derived stem cells of obese patients were found to be impaired in their angiogenic potential since they carry a reduced content of VEGF, MMP-2, and miR-126 [70].

In addition to the observation that circulating EV number (including adipocyte- and hepatocyte-derived EV) was significantly higher in men than in women and that EV number correlated significantly with triglycerides and HOMA-β [50], Rigamonti et al. [71] suggest the intriguing possibility to modulate the various types of EV by exercise in a sex-specific manner. Indeed, after exercise, the circulating levels of microvesicles were higher, while those of exosomes were lower in females than in males. Additionally, data of a relatively small study suggest that sex underpins differential EV responses to exercise in obese sedentary young adults [72]. Comparing the effects on EV of interval exercise or high-intensity continuous exercise, it has been observed that both kinds of exercises can lower EV counts (CD31^+^/CD42b^−^) in men, but not in females. Yet, high-intensity continuous exercise increases the number of endothelial-derived large EV (CD62E^+^) in females [72].

In conclusion, accumulating evidence suggests a potential involvement of EV in promoting inflammation in obesity-associated insulin resistance, although the mechanisms leading to enhanced levels of EV and their biological effects in obesity have yet to be fully clarified. Importantly, interventions, such as exercise, may differently modify the EV characteristics in overweight/obese males and females. Whether EV acquire anti-inflammatory properties after exercise is unknown.

### 4.2. EV and Diabetes

Several studies suggest an important role of abnormal EV in the etiology of diabetes mellitus and related cardiovascular disease [1,46,56,73,74].

It has been suggested that, depending on their cellular origin, EV can contribute differently to the progression of the disease. Indeed, increased exosome plasma level and changes in its content, in particular both up- or down-regulation of miRNA cargo, have been reported in diabetes patients [24,70,73,75]. It has been suggested that abnormal EV from different cells could induce insulin resistance through activating inflammation, affecting the insulin receptor and down-regulating glucose transporter type 4 (GLUT-4) [74].

Higher numbers of MP released from platelets, monocytes, and endothelial cells have been detected in plasma from patients with Type 1 diabetes mellitus (T1DM) in comparison with Type 2 (T2DM) and healthy subjects [76]. Dysregulation of coagulation related to MP and contributing to greater sex-dependent cardiovascular risk has been described. The reduction in the number of MP after aspirin administration and the antithrombotic effect of statins observed in patients with T1DM suggest their platelet origin and their role in the progression of the disease [77]. The increased number of platelet-released MP detected in the plasma of young T1DM patients compared with healthy subjects suggested a correlation with the incidence of microvascular complications commonly found in these patients [78]. Although the BMI for the T1DM patients was not reported in these studies, it has been shown that the levels and cargo of endothelium- and platelet-derived MP from T1DM patients with normal weight (BMI < 25) result in higher procoagulant activity, suggesting that overweight or obesity may be independent elements altering the biological effects of MP [79].

Exosomes released from pancreatic β-cells carry autoantigens that may participate in the generation of the autoimmune response leading to β-cell dysfunction in T1DM [80]. Moreover, several other factors, including miRNAs, may be involved in exosome or exosome-like vesicle-mediated diabetes nephropathy [81]. Some reports, however, have shown that both Exo and MV could also reduce the inflammatory processes. For instance, Exo present in the urine of T1DM patients have a miRNA cargo that could be protective for diabetic glomerulopathy [75], and microvesicles from a human mesenchymal stem cell line exert an anti-inflammatory effect in mononuclear blood cells isolated from T1DM patients [82]. In these studies, the sex of T1DM patients was not considered, although the difference in circulating EV between men and women may be directly due to sex and/or to the different degree of metabolic stress related to sex [50].

Several studies suggest the involvement of MP released by platelets, monocytes, and endothelial cells in T2DM [83]. It has been suggested that miRNAs are the most relevant EV cargo involved in the pathogenesis of T2DM, particularly in patients with a higher risk of developing coronary artery disease [84,85]. Indeed, up-regulated Exo miRNA cargo (miR-15b, miR-34a, and miR-636) [84] and reduced levels of miR-126 and miR-26a in circulating MP of diabetic patients compared to non-diabetic patients [85] were described. In addition to miRNA alterations, in T2DM patients, a reduced adiponectin plasma level, which, in turn, may result in the inactivation of adenosine monophosphate-dependent protein kinase was described [86]. However, proteins such as aquaporins [81] and insulin receptor substrate 1 were also detected in urine-derived Exo from T2DM patients [87].

The increased number of plasma EV derived from platelets [88], monocytes, and endothelium [76] in overweight and obese (BMI between 26 and 47) T2DM patients suggests their ability to lead to a procoagulant state and to increase the severity of the disease. Interestingly, a controlled diet may modulate the content of tissue factor, fibrinogen, and P-selectin in the platelet-microvesicle cargo of patients with T2DM [88].

EV may participate in the development and progression of cardiac damage in diabetic cardiomyopathy by promoting an abnormal crosstalk between endothelial cells and cardiomyocytes. The higher levels of miR-320 found in EV derived from cardiomyocytes of diabetic rats and its transfer to endothelial cells may inhibit their proliferation, migration, and tube formation by down-regulating the expression of insulin-like growth factor-1 (IGF-1), heat shock protein (HSP)20, and the transcription factor2 (Ets2). HSP70 in platelet-derived EV from diabetic rats lacks the ability to activate the extracellular signal-regulated kinase 1/2 (ERK1/2) and HSP27 cardioprotective pathways in injured cardiomyocytes. Moreover, the macrophage stimulating 1 (Mst1) protein in EV from cardiac endothelial cells of diabetic mice can be transferred to cardiomyocytes and inhibits autophagy, promotes apoptosis, and suppresses glucose metabolism in these cells [74]. In Table 3 of the article by Xiao et al. [74], the application of EV in the therapy of T2DM and its complications (e.g., nephropathy, cardiomyopathy and retinopathy) are summarized.

Depending on their content, exosomes may also exert a protective effect in diabetes. While an impaired regulation of exosome-mediated cross-talk between liver, adipose tissue, and skeletal muscle participates in the functional alterations associated with diabetes and obesity [58], factors called *exerkines*, released in exosomes in response to endurance exercise, play an important role in mediating the systemic benefits of exercise in diabetic or obese patients. It is necessary to elucidate the mechanisms by which sex may induce differential EV responses to exercise to identify specific exercises potentially exploitable to treat and/or prevent disease [89,90,91]. Interestingly, it has been reported that stem/progenitor cell-derived EV may be used to treat T2DM and its complications in animal studies (reviewed in [74]).

Two studies have reported that EV, by transferring miRNAs, can be used to ameliorate erectile dysfunction in diabetic rat models [92,93]. RNA sequencing revealed that EV were enriched for distinct classes of proangiogenic miRNA (miR-21-5p, let-7 family, miR-10 family, miR-30 family, and miR-148a-3p) [93]. Yet, it has been reported that hyperglycaemia can lead to matrix and collagen type IV production through STAT5A and miR-21 up-regulation. Thanks to the release of EV cargo, such as miR-222, which post-transcriptionally regulates STAT5A controlling miR-21 expression, mesangial cells treated with EV are protected from fibrogenic signals. In this setting, EV treatment also saves mesangial cells from harmful mitochondrial signals by up-regulating the expression of CoxIV (a nuclear encoded mitochondrial electron transport chain component) [70].

In conclusion, accumulating evidence supports the potential role of EV in the pathogenesis of diabetes, especially T2DM, by inducing insulin resistance and inflammation, down-regulating GLUT-4, and modulating the expression of insulin receptors. EV can also participate in the development of diabetic complications. Although their precise role is still unclear, the effects of EV are ascribed to several abnormal molecules carried, including proteins and miRNAs. On the other hand, miRNAs derived from EV that may induce beneficial effects in some diabetic complications and sex differences on cargo compositions are starting to emerge [70,92,93].

### 4.3. EV, Hypertension and Sex

Systemic hypertension is a chronic condition characterised by persistently elevated blood pressure in the arteries and represents a significant risk factor for ischaemic diseases [94,95,96].

Both in women and in men, the development of hypertension is correlated with diabetes and metabolic syndromes [97]. Clearly, the metabolic syndrome accelerates the arterial aging and amplifies hypertension-related cardiac and renal alterations. Some features of the metabolic syndrome, when considered individually, may have little or no influence on damage of target organs, but when associated with hypertension, favour the development of microalbuminuria, aortic stiffness, as well as cardiac hypertrophy and dysfunction. Hypertensive patients are usually characterised by a multifactorial disease, and are at risk of developing a “cardio-renal syndrome” (CRS) if the heart or kidney damage leads to the dysfunction of the other organs [98,99,100]. Moreover, a strict association between hypertension and endothelial dysfunction has been reported [101,102]. Indeed, hypertension, CRS, and endothelial dysfunction were found to be associated with EV alteration (see below).

It has been shown that endothelial-derived MP directly promote vascular inflammation and induce dysregulation of coagulation, contributing to greater sex-dependent cardiovascular risk, due to the impact of hormones on arterial function. In a clinical study, an association between circulating endothelial-derived MP levels and hypertension was observed in both hypertensive men and women without other cardiovascular diseases [45]. Similarly, a relationship between hypertension and an increase in endothelial- and platelet-derived MP was found in patients with severe uncontrolled hypertension [103]. Recently, endothelial MP, *endocan*, and *endoglin* have been proposed as novel biomarkers of endothelial dysfunction [104]. In Table 1 of the article by Leite et al. [104], the quantification and phenotype of endothelial MP in different diseases are reported. In particular, endothelial MP results are strongly correlated with both diastolic and systolic hypertensions.

It has been shown that patients with chronic thromboembolic pulmonary hypertension are also characterised by a significant increase in *endoglin^+^* endothelial MP compared to healthy controls. These data have suggested that *endoglin^+^* endothelial MP may represent markers of dysfunction or, alternatively, a protective factor which attempts to counteract the effects of vascular occlusion and endothelial damage [105,106].

#### EV as a Therapeutic Tool in Hypertension

Studies investigating the potential contribution of EV as therapeutics in metabolic dysfunction are limited; however, data are emerging on their use as potential therapeutic agents for different types of hypertension and their associated complications (for reviews see [107,108]). Recently, EV have been proposed as effective therapeutic options for both hypertension and CRS in animal studies. In particular, adipose-derived mesenchymal stromal cells (ASC-EV) administered in multiple doses can be effective as a therapy for the treatment of hypertension and CRS, preventing chronic kidney disease (CKD). Moreover, Lindoso et al. [106] demonstrated that ASC-EV containing miRNAs, in particular those belonging to the miR-200 family, play a pivotal role in CKD progression toward CRS, and could be a therapeutic target in hypertensive-induced CKD. Briefly, EV obtained by ASC and administered in a hypertensive animal model were found to be protective in preserving kidney filtration and in protecting the kidney from damage. Such EV are able to induce relevant anti-inflammatory effects as they reduce the expression of *Monocyte Chemoattractant Protein-1* (MCP-1) and *Plasminogen Activating Inhibitor-1* (PAI1) and the recruitment of macrophages in the kidney. In the cardiovascular system, EV prevented the cardiac fibrosis and kept the blood pressure in the normal range. These protective effects are likely due to their miRNA cargo, in particular miR-200 [106].

Several critical issues must be addressed, and more effort in EV research including more in vivo models and translational approaches is needed before EV may have a therapeutic application in humans [1,28,30,109,110].

## 5. EV, Metabolic Syndrome, and Sex Interaction

Although vascular inflammation plays a central role in coronary artery disease and associated comorbidities; sex differences have been described in preclinical and experimental medicine studies in this scenario [14]; and EV, including Exo, may have pro-inflammatory [63,68,69] or anti-inflammatory [75,82,106] properties, it appears that only a few studies have considered sex in observing EV in these comorbidities (see above and Table 1).

Here, we underline some studies that attempted to analyse metabolic syndrome and sex interactions. For instance, Amabile et al. [45], in a retrospective analysis, associated the levels of circulating endothelial MP with cardiometabolic risk factors, particularly dyslipidaemia, in both sexes. Moreover, Kranendonk et al. [67] evidenced that sex influences body fat distribution and performed an analysis for sex disaggregated data between adipose tissue distribution and EV-markers. These authors, in agreement with Karastergiou et al. [43], observed that males are more likely to develop abdominal obesity and display a higher incidence of metabolic and cardiovascular disease. In these two studies, no interaction for sex was demonstrated in the comparison between metabolic parameters and EV-markers. However, in patients with evident vascular disease, EV enriched in *cystatin C* was positively correlated with the metabolic complications of obesity. In contrast, EV-CD14 level was inversely related to visceral obesity only in males and was associated with a relative risk reduction for the generation of T2DM [67].

Kobayashi et al. [50] and Eguchi et al. [51] proved that metabolic stress is more severe in men and that the elevated number of circulating EV is associated with the increased levels of insulin and Homeostatic Model Assessment for Insulin Resistance (HOMA-IR). This suggests that the differences in the number of circulating EV between men and women should be likely due to the difference in sex as well as the degree of metabolic stress, also influenced by the sex difference. However, a larger scale of investigation is needed to confirm the lower EV number in diabetic men compared with healthy and unhealthy women.

Recently, Rigamonti et al. [71] suggested that a single bout of acute exercise can modulate the release of EV in the circulatory system in a tissue- and sex-dependent manner, suggesting that exercise-related benefits may depend on an interplay of endocrine and metabolic factors in various tissues, thus influencing the formation of EV.

Overall, studies described in this paragraph and those described in the previous paragraphs suggest that differences in EV number, origin, and composition can be detected and can be influenced by sex. In particular, the studies by Durrer et al. [72] and Rigamonti et al. [71] also suggested that monitoring EV in a sex-specific manner may be useful to understand the beneficial effects of physical activity in patients with metabolic syndrome. Therefore, sex is a confounding factor in these clinical and experimental conditions that cannot be ignored anymore. Several recent studies underlined a clear sex difference in the resolution of inflammation (reviewed in [14]). Since EV may be exploitable for inflammation resolution [75,82,106], it is mandatory to study sex differences in EV properties in all pre-clinical and clinical conditions.

**Table 1:** Selection of studies considering EV and individual cardiovascular risk factors of MS (obesity, diabetes, and hypertension): possible role of sex.

Main Disease and Model	EV Origin	Main Conclusions	Participants and Sex Role	Ref
Obesity				
Humans	PLT and ECs	In women with severe obesity, platelet, and endothelial MP are increased independently of individual cardiovascular risk factors or clustered as MS.	151 obese and 60 lean; all women.	[53]
Humans	Circulating ACs—and hepatocytes	Circulating EV number was significantly higher in men than in women and EV number correlated significantly with triglycerides and HOMA-β.	203 participants (127 women). Sex role: yes.	[50]
Humans	ECs	Different types of exercises can affect EV counts (lower CD31^+^/CD42b^−^) in men, but not in females. High-intensity exercise increases the number of endothelial-derived EV (CD62E^+^) in females.	13 obese (6 males, 7 females). Sex role: NC	[72]
Humans	ACs	Visceral adipocyte sheds exosomes containing miRNAs capable of regulating end-organ TGF-β and Wnt/β-catenin signalling.	7 obese and 5 lean subjects; all females	[63]
Humans	PLTs, LCs and ECs	Decrease in circulating MP in obese patients 3 and 12 months after sleeve gastrectomy. The decrease in MP is positively correlated with the decrease in BMI.	20 obese; 10 males and 10 females. Sex role: NC	[62]
Humans and mice	PLTs, ACs, and ECs	EV specific carriers of MIF involved in the development of insulin-resistance associated with diabetes and obesity.	34 obese; 16 males. Sex role: NC	[61]
Humans	Plasma (PLTs and skeletal muscle)	After exercise circulating levels of MV and exosomes were, respectively, higher and lower in females than males.	15 obese (8 males) and 8 (4 males) lean subjects. Sex role: NC	[71]
Humans EPCs	ACs	Obese EV display reduced content of VEGF, MMP-2, and miR-126 and show impaired angiogenic potential compared with normal EV under hyperglycaemia.	10 obese and 6 non-obese participants. Sex role: NC	[70]
Diabetes				
Humans	PLTs	PMPs are elevated in children and adolescents with T1DM and can be considered as an early marker of microvascular complications and subclinical atherosclerosis.	8 among children and adolescents. Sex role: NC	[78]
Humans	PLTs, MCs and ECs	Aspirin therapy inhibits vascular wall cell activation and MP shedding similarly in T1 and T2DM, in which circulating MP were higher in patients respect to healthy subjects.	43 diabetic patients; 24 males. Sex role: NC	[76]
Humans and studies in vitro	PLTs	In T1DM, atorvastatin treatment and platelet-derived MP influence the fibrin network formation in vitro: fibrin network permeability decreased if MP were added to normal plasma.	20 diabetic patients. Sex role: NC	[77]
Humans and Rats in vitro	Islet cells, and rat cell line INS-1E	Exosomes release induced by stress play a role in the initiation of autoimmune responses leading to β-cell dysfunction and in T1DM.	Numbers: NS. Sex role: NC	[80]
Humans and Mice	Mesangial cells	Urinary exosomal miRNA content is altered by T1DM and complicated by nephropathy. In particular, miR-145 is proposed as a biomarker/player in the complication.	12 diabetic patients and 30 diabetic mice; all males.	[75]
Diabetes Humans	PLTs	Levels of circulating PMPs are correlated with T2DM and may have procoagulant effects contributing to the pathogenesis of thrombosis, inflammation and atherosclerosis.	10 diabetic patients; 8 males. Sex role: NC	[88]
Hypertension				
Humans	PLTs and ECs	Endothelial- and platelet-derived MP are correlated with the level of both systolic and diastolic blood pressures.	43 (29 males) hyper and 16 (13 males) normotensive	[103]
Humans	ECs and PLTs	Circulating endothelial MsP levels were associated with the presence of dyslipidaemia and hypertension. Two endothelial MP sub-populations (CD144^+^ or CD31^+^CD41^-^) with regard to sex and hypertension are described.	844 (363 males). Sex role: several differences are reported	[45]
Human ASCs and Rats	ASC	Administration of human ASC-EV limits renal damage, inflammation, and kidney fibrosis in a hypertensive-induced CKD murine model.	8-13 male rats for group	[106]

**Abbreviations:** ACs = adipocytes; ASC = adipose-derived mesenchymal stromal cells; BMI = body mass index; ECs = endothelial cells; EPCs = endothelial progenitor cells; EV = extracellular vesicles; LCs = leucocytes; MCS = monocytes; MP = microparticles; MV = microvesicles; MMP-2 = matrix metalloproteinase 2; MS = metabolic syndrome; NC = not considered endothelial cells; NS = not specified; PLTs = platelets; PMPs = platelet-derived microparticles; T1DM = Type 1 diabetes mellitus; T2DM = Type 2 diabetes mellitus; VEGF = vascular endothelial growth factor (VEGF).

## 6. Conclusions and Perspectives

Recently, an important predictive and protective role of EV was proposed in many pathophysiological conditions. The ability of EV to carry and transfer their cargo, proteins, lipids, amino-acids, mRNAs, and miRNAs can be exploited as a promising biomarker of comorbidities and also as a therapeutic tool in pro-ischaemic conditions. Cardiovascular diseases and in particular myocardial ischaemic disease are correlated with chronic comorbidities, including metabolic alterations (e.g., diabetes, obesity) and hypertension, which can be part of the metabolic syndrome. In this scenario, it is likely that important sex-related differences may significantly affect EV number, composition, and origin. To date, very few studies have considered sex differences in EV obtained from patients with comorbidities predisposing to ischaemic diseases. Nevertheless, sex differences are emerging in EV composition in these conditions. Yet, we are still far from solving several key issues to translate EV into clinical use. These include the best choice of cell, their scalability, and more importantly their safety. Rigorous and reproducible preclinical and clinical studies are of utmost importance to test hypotheses on sex differences in cardiovascular research [109,110]. Undoubtedly, the basic science must be revolutionary in concepts and in suggesting new, exciting hypotheses, but we must ensure safe and efficient therapies for the specific diseases of patients: new translational and clinical studies are mandatory to confirm the contribute of EV as early disease biomarkers and their suitability as therapeutic tools. Of course, the biological origin of EV makes it particularly necessary that future studies take into consideration sex-related differences in addition to the comorbidities associated with CVDs.

## Data Availability

Not applicable.
